# Airway and Extracellular Matrix Mechanics in COPD

**DOI:** 10.3389/fphys.2015.00346

**Published:** 2015-12-02

**Authors:** Cécile M. Bidan, Annemiek C. Veldsink, Herman Meurs, Reinoud Gosens

**Affiliations:** ^1^Department of Molecular Pharmacology, University of GroningenGroningen, Netherlands; ^2^Groningen Research Institute for Asthma and COPD, University of GroningenNetherlands; ^3^Laboratoire Interdisciplinaire de Physique (LIPhy), Université Grenoble AlpesGrenoble, France; ^4^Centre National de la Recherche Scientifique, LIPhyGrenoble, France

**Keywords:** COPD, extracellular matrix proteins, airway obstruction, mechanics, collagen, elastin, proteoglycans, emphysema

## Abstract

Chronic obstructive pulmonary disease (COPD) is one of the most common lung diseases worldwide, and is characterized by airflow obstruction that is not fully reversible with treatment. Even though airflow obstruction is caused by airway smooth muscle contraction, the extent of airway narrowing depends on a range of other structural and functional determinants that impact on active and passive tissue mechanics. Cells and extracellular matrix in the airway and parenchymal compartments respond both passively and actively to the mechanical stimulation induced by smooth muscle contraction. In this review, we summarize the factors that regulate airway narrowing and provide insight into the relative contributions of different constituents of the extracellular matrix and their biomechanical impact on airway obstruction. We then review the changes in extracellular matrix composition in the airway and parenchymal compartments at different stages of COPD, and finally discuss how these changes impact airway narrowing and the development of airway hyperresponsiveness. Finally, we position these data in the context of therapeutic research focused on defective tissue repair. As a conclusion, we propose that future works should primarily target mild or early COPD, prior to the widespread structural changes in the alveolar compartment that are more characteristic of severe COPD.

## Introduction

Chronic obstructive pulmonary disease (COPD) is one of the most common lung diseases worldwide, and is a consequence of (second-hand) exposure to tobacco smoke and/or environmental pollutants. In 2012 it caused 6% of all deaths (World Health Organization, 2012) and the burden of disease is increasing rapidly; currently affecting over 200 million people. The WHO estimates that COPD will become the third leading cause of death in westernized countries by 2020. COPD is characterized by a progressive loss of lung function with airflow obstruction that is not fully reversible with bronchodilators (Rabe et al., 2007). Airflow obstruction is determined as a reduction in the ratio of forced expiratory volume in 1 s (FEV1) over forced vital capacity (FVC). FEV1 represents the volume of air an individual can expire with force within 1 s following a deep inspiration, whereas FVC represents the total volume of air an individual can expire with force following a deep inspiration. An FEV1/FVC ratio lower than 70% is considered a hallmark of COPD, progresses with time, and is the principal determinant of disease severity in the GOLD I-IV classification criteria (Rabe et al., 2007).

Airflow obstruction in COPD is caused by a combination of factors, including pulmonary inflammation associated with bronchitis and mucus hypersecretion, small airways remodeling and lung emphysema in varying combinations and severities (Postma and Timens, 2006). Unfortunately, no current drug treatments modify the course of the disease. Although chronic inflammation of the peripheral airways and lung parenchyma contributes to progressive obstruction of the airways, anti-inflammatory glucocorticosteroids are largely ineffective in inhibiting lung function decline (Barnes, 2013). Bronchodilators are the mainstay of COPD treatment, of which long-acting β_2_ agonists, long-acting anticholinergics and combinations hereof have become the standard of care (Meurs et al., 2013). Both classes of drugs act primarily on the airway smooth muscle, reducing bronchoconstriction and therefore airflow obstruction in a clinically meaningful way. Their effectiveness is however also restricted, as even the most recently introduced combination therapies of long-acting β_2_ agonists and long-acting anticholinergics produce an improvement in FEV_1_ in the range of ~150–200 mL (Vogelmeier et al., 2013; Buhl et al., 2015), which is clearly clinically significant, but represents an improvement in the FEV1/FVC ratio of around 5 percentage points only. Clearly, airway smooth muscle contraction is an important, but not the main contributor to lung function decline in patients with severe COPD. In this review, we summarize the factors that regulate airway narrowing and provide insight into the relative contributions of different constituents of the extracellular matrix and their biomechanical impact on airway obstruction. We then review the changes in extracellular matrix composition in the airway and parenchymal compartments at different stages of COPD, and finally discuss on how these changes impact airway narrowing and the development of airway hyperresponsiveness. Finally, we position these data in the context of therapeutic research focused on defective tissue repair. As a conclusion, we propose that future works should primarily target mild or early COPD, prior to the widespread structural changes in the alveolar compartment that are more characteristic of severe COPD.

### Airway mechanics in different structural compartments

Even though bronchoconstriction is triggered by airway smooth muscle contraction, the extent of airway narrowing depends on a range of other structural and functional determinants that impact on active and passive tissue mechanics (Suki and Bates, 2008, 2011; Lauzon et al., 2012). The mechanical events occurring in the lung are (i) the cyclic breathing imposed by the diaphragm on the whole lung, (ii) the bronchoconstriction initiated by the active contraction of the airway smooth muscle (ASM) and (iii) the resulting stretching of the extracellular matrix (ECM) and of the other cells that surround the airways (Politi et al., 2010; Lauzon et al., 2012). These cells and ECM will respond both passively and actively to the mechanical stimulation induced through their intrinsic mechanical properties and via mechanotransduction pathways (An et al., 2007). Therefore, a proper understanding of lung mechanics in COPD needs to consider the role of the cells and their ECM in the different structural compartments of the lung, as well as their interplay (Suki and Bates, 2008, 2011; Lauzon et al., 2012). Diverse experimental models have been developed to address this topic at different scales (Wright et al., 2013).

### Extracellular matrix mechanics

The ECM is defined as a protein network mainly composed of compliant elastin, stiff collagen and proteoglycans, all secreted and organized by the cells embedded within this matrix (Dekkers et al., 2009; Humphrey et al., 2014). It is a non-linear elastic material that becomes stiffer when stretched (Storm et al., 2005; Erk et al., 2010; Hiorns et al., 2014), but it is also a viscoelastic material that relaxes or creeps under constant deformation or load respectively (Faffe and Zin, 2009). These passive responses are highly heterogeneous since they mainly depend on the structural and mechanical properties of the collagenous network (Shayegan and Forde, 2013), which in turn depends on the presence of proteoglycans such as decorin (Pins et al., 1997). In the lungs, the ECM in the close vicinity of the ASM cells is dense and structurally different from the ECM that constitutes the alveolar tissue outside the airway, i.e., the parenchyma (Stamenovic et al., 2004).

### The airway compartment

The airway compartment consists of the airway epithelium, the reticular basement membrane, the subepithelial region, the ASM layer and an adventitial region, which in larger airways contains cartilage. The ASM cells can generate forces in response to biochemical stimuli. The amount of force generated by each cell is essentially determined by the amount of actomyosin fibers in its cytoskeleton. This force is transmitted to the surroundings through an actin network and adhesion complexes, which densify and mature upon mechanical stimulation (Brenner, 1991; Gunst and Zhang, 2008). The ability for the ASM cells to contract and relax has been widely studied using *in vitro* cell cultures. Single ASM cells plated on soft gels and stimulated with contractile agonists exert tractions up to 200Pa (force normalized by the area of application) on their substrate (Tolic-Norrelykke et al., 2002). Such traction force microscopy (TFM) studies also show that the forces applied by a cell on its surroundings are not isotropic (not the same in every directions).

**Figure 1:**
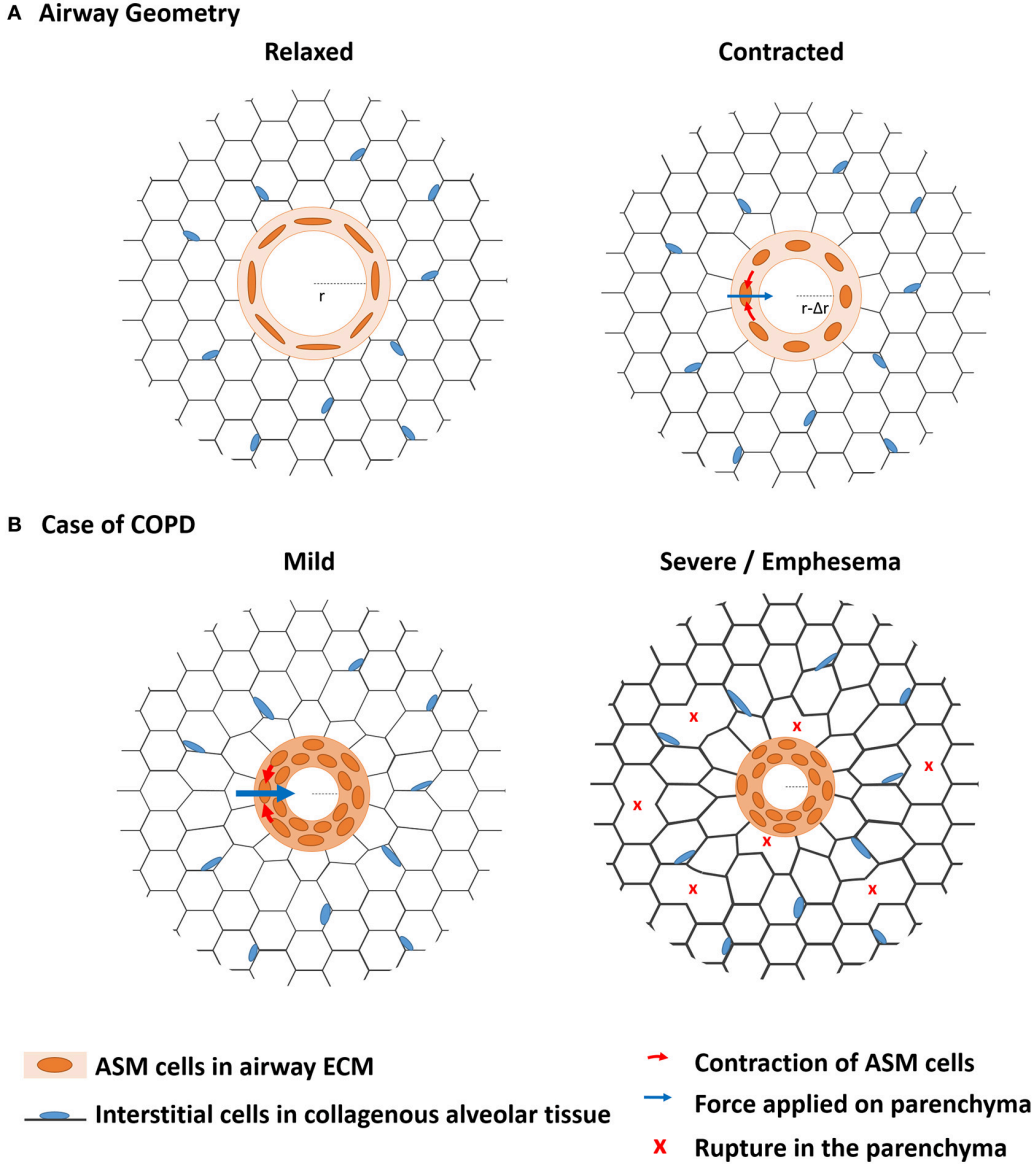
**The biomechanics from bronchoconstriction to COPD**. **(A)** The contractile airway smooth muscle (ASM) is embedded in an alveolar tissue called parenchyma. When they contract in reaction to external stimuli, the ASM cells aligned along the perimeter of the airway apply a radial force on the parenchyma, which is deformed. In healthy lungs, the resulting airway narrowing is transient and fully reversible. **(B)** In case of COPD, chronic inflammation leads to chronic airway hyperresponsiveness (AHR). Airway narrowing is more profound than in healthy lungs so that the ASM gets thicker by cell hypertrophy and ECM remodeling, which prevents full reversibility of airway narrowing. The resulting sustained load expands the remodeling processes to the parenchyma, which mechanical properties are affected up to disruption of the alveolar walls. Emphysema is particularly characteristic of severe COPD.

As far as mechanics is concerned, it is not only important to focus on the ASM contractile cells that generate the driving forces of bronchoconstriction, but it is also critical to characterize the structural components on which these cells adhere, and that withstand and transmit the forces across the lungs (An et al., 2007; Khan et al., 2007, 2010). Because the ASM cells are aligned around the airway with only a small angle deviating from the transversal plane (Lei et al., 1997), their contraction results in a circumferential force (Figure 1A). The orientation of the ASM cells in the airway wall therefore determines the magnitude of the resulting force applied during contraction and thus the deformation of the airway as a whole (Bates and Martin, 1990; Lei et al., 1997). This contraction leads to a shortening of the perimeter (Δ*P* = 2π.Δr) or narrowing of the airway (Δr), also called bronchoconstriction (Bates and Martin, 1990; Figure 1A). In a healthy lung, deep inspirations help the same cells to relax so that the airway dilates to recover its initial diameter after a few breathing cycles (Lavoie et al., 2012; West et al., 2012).

### The parenchymal compartment

The parenchymal compartment defines the alveolar tissue made of interstitial cells and a large proportion of ECM, and which surrounds the airways. The parenchymal tissue has a major impact on airway mechanics due to its role in the tethering of forces via the parenchymal attachments that in the end link chest movements during inspiration to airway opening (Suki and Bates, 2008, 2011; Lauzon et al., 2012). Parenchymal tethering imposes an additional dynamic load on the airway smooth muscle, which impacts on airway narrowing and supports the relaxant effect of a deep inspiration (An et al., 2007). The importance of the parenchymal lung tissue for airway mechanics was recently demonstrated in a lung slice model of small airway narrowing following digestion of elastin and/or collagen fibers (Khan et al., 2007, 2010). This shows that the extracellular matrix has a key role in connecting the parenchymal compartment to the airway compartment, and in affecting airway mechanics.

### Interplay between the airway and parenchymal compartment: role of the ECM

ASM contraction imposes a deformation of the parenchyma by a distance of Δr along the radial direction (Figure 1A). The forces involved in this deformation propagate through the parenchyma, which mechanical response is mainly controlled by the parenchymal ECM (Yuan et al., 1997; Cavalcante et al., 2005). The local stiffness of the parenchymal segments and junctions measured in decellularized lung tissues was shown to be rather homogeneous but to increase close along the pleura (Luque et al., 2013). The heterogeneities of the mechanical cues sensed by the cells are thus mainly attributed to the heterogeneity of the local geometries and architectures of the alveolar walls.

Moreover, intimate biomechanical interplays exist between ASM cells and ECM (Suki and Bates, 2008; Zhang and Gunst, 2008; Dekkers et al., 2009). Indeed, the ECM on which cells adhere is a dynamic material with mechanical properties that are passively and actively adapted to the forces it undergoes. The process of mechanotransduction leads the mechanical properties of the microenvironment to control a lot of cellular cues such as differentiation (Huang et al., 2012), proliferation, the cellular compliance to deformation/relaxation (Stamenovic et al., 2004), but also ECM maintenance (Humphrey et al., 2014). The collagen, elastin and proteoglycans contents as well as the organization of the ECM are therefore continuously actively adjusted by the cells so as to match the requirements of the mechanical environment and ensure tissue integrity.

Because the ECM plays key roles in force transmission throughout the tissue, changes in ECM composition in COPD may impact on the association between ASM cell shortening on the one hand and airflow obstruction defined as a reduction in the FEV1/FVC ratio on the other.

## Extracellular matrix changes in COPD airways and lung parenchyma

In view of the key role of the ECM in determining airway narrowing, it is of importance to consider the changes in ECM expression in COPD. The altered expression of ECM proteins is one of the pathological hallmarks characterizing the progression of COPD and contributes to remodeling the airways and the parenchyma. Of particular interest is the relationship between COPD severity and ECM protein expression as in severe COPD, parenchymal tissue damage (emphysema) is more profound, whereas in mild to moderate COPD, emphysema is milder on average (Wang et al., 2015). It is therefore important to make an inventory of the changes in ECM composition in mild to moderate COPD and to question whether these changes in tissue integrity are sufficient to induce changes in airway narrowing, even in patients without obvious emphysema. This discussion is also relevant to early COPD, although little data is available as to the ECM expression characteristics in early COPD. Although not a main focus in this review, this discussion may even be relevant to healthy smokers as a subpopulation of healthy smokers of around 27% has emphysema revealed by computed tomography in comparison to 1.1% of healthy non-smokers (Hoffman et al., 2014; Smith et al., 2014)

Various studies have shown altered expressions of major ECM components such as elastin, collagen and proteoglycans in COPD, compared to healthy smokers or healthy controls (Table 1). Those studies also indicate differences in the levels of ECM protein dependent on the COPD stage, showing that remodeling is already present in mild or moderate COPD, but progresses to more significant pathology in severe disease (Hogg and Timens, 2009). Some proteins exhibit specific changes in expression dependent on location, e.g., small airways or parenchymal tissue. A summary of the main changes in ECM protein expression in COPD according to severity and location is provided in Table 1.

**Table 1:** **Extracellular matrix changes in airways and parenchyma of patients with COPD**.

**ECM protein**	**Mild/moderate COPD Small airways**		**Mild/moderate COPD Parenchyma**		**Severe COPD Small airways**		**Severe COPD Parenchyma**	
		**References**		**References**		**References**		**References**
Elastin	↓	Eurlings et al., 2014	↓ ↓ ↔	Eurlings et al., 2014 Merrilees et al., 2008 Annoni et al., 2012	↓	Eurlings et al., 2014	↓ ↑ ↑	Eurlings et al., 2014 Vlahovic et al., 1999 Deslee et al., 2009
Biglycan	↔	Annoni et al., 2012	↔	Annoni et al., 2012	↓	van Straaten et al., 1999	↓	van Straaten et al., 1999
Decorin	↔	Annoni et al., 2012	↔	Annoni et al., 2012	↓ ↓	Zandvoort et al., 2006 van Straaten et al., 1999	↓ ↓	Zandvoort et al., 2006 van Straaten et al., 1999
Versican	↔	Annoni et al., 2012	↑ ↓	Merrilees et al., 2008 Annoni et al., 2012	↑	Hallgren et al., 2010	↑	Hallgren et al., 2010
Total Collagen	↑	Eurlings et al., 2014	↑	Eurlings et al., 2014	↑	Eurlings et al., 2014	↑	Eurlings et al., 2014
Collagen I	↓ ↑ ↑	Annoni et al., 2012 Harju et al., 2010 Kranenburg et al., 2006	↓	Annoni et al., 2012	↓	Harju et al., 2010		
Collagen III	↓ ↔	Harju et al., 2010 Annoni et al., 2012	↔	Annoni et al., 2012	↓	Harju et al., 2010		
Collagen IV	↔	Annoni et al., 2012	↔	Annoni et al., 2012				
Tenascin	↑ ↑ ↑	Annoni et al., 2012 Lofdahl et al., 2011 Liesker et al., 2009	↔	Annoni et al., 2012				
Fibronectin	↑	Annoni et al., 2012	↔	Annoni et al., 2012	↓	Gosselink et al., 2010		
Hyaluronan	↑ ↑	Eurlings et al., 2014 Dentener et al., 2005	↑	Eurlings et al., 2014	↑	Eurlings et al., 2014	↑	Eurlings et al., 2014
Laminin	↔ ↔ ↑	van Straaten et al., 1999 Liesker et al., 2009 Kranenburg et al., 2006						

### Elastic fibers

An important finding is the decrease in the protein expression of elastin, the volume fraction of which is reduced in both airways and alveoli of patients with COPD in comparison to controls (Black et al., 2008). This reduction appears similar in both mild to moderate and severe COPD airways (Eurlings et al., 2014). The expression of elastin in the parenchyma was also found reduced in patients with mild to moderate and severe COPD in that study (Eurlings et al., 2014) and by others in mild to moderate COPD (Merrilees et al., 2008). However, some have reported increased expression of elastin in the remaining alveolar walls in patients with severe COPD (Vlahovic et al., 1999). Furthermore, in patients with mild to moderate COPD, Annoni et al. report no changes in elastic fiber content in comparison to healthy subjects, whereas elastic fiber content in these patients is reduced in comparison to healthy smokers (Annoni et al., 2012). Interestingly, Deslee et al. showed that the elastin fibers in the alveolar walls of patients with severe COPD are considerably less densely packed, unraveled and loose in comparison to healthy subjects (Deslee et al., 2009). These changes occur in spite of increased elastin mRNA expression in patients with severe COPD, indicating inadequate attempts to activate a repair process in these patients. Thus, whereas the available reports are disconcordant in terms of total elastin expression in the parenchyma in patients with COPD, possibly the elastin fibers present are less well organized and looser in comparison to healthy controls, contributing to the progressive loss of elastic recoil in small airways and parenchyma in patients with COPD. These changes in elastin are relevant to both mild to moderate and severe COPD, although clearly more profound in severe COPD.

### Proteoglycans

A similar decline in protein levels is found for the proteoglycans biglycan and decorin in the peribronchial region and parenchyma of patients with severe COPD (van Straaten et al., 1999; Zandvoort et al., 2006). The loss of decorin expression in the parenchyma correlated positively to COPD severity and paralleled a decrease in abundance of TGF-β_1_ in the patients with severe COPD (Zandvoort et al., 2006). A trend for reduced expression of biglycan in parenchyma of patients with mild to moderate COPD compared to non-smokers and a significant reduction in decorin expression in parenchyma of patients with COPD in comparison with non-smokers has also been reported (Annoni et al., 2012). This suggests that alterations in the abundance of these proteoglycans are more pronounced in severe COPD.

In contrast to biglycan and decorin, the proteoglycan versican was found increased in mild to moderate COPD, inversely correlating to the elastic fiber content (Merrilees et al., 2008). This fits with findings showing increased versican production by fibroblasts obtained from COPD patients in comparison to those obtained from non-smoking controls (Hallgren et al., 2010). High levels of versican may contribute to the loss of elastic fibers during progression of COPD as versican inhibits synthesis and regeneration of these fibers (Merrilees et al., 2008). Nonetheless Annoni et al. (2012) found no changes in versican content in the airway compartment and even somewhat reduced versican levels in mild to moderate COPD patients in comparison to non-smoking controls. The reason for this apparent discrepancy is not clear, but can be related to the different methods of analyses used in both studies.

### Collagens

For collagen the studies are inconsistent. Increased total collagen expression in the airway and parenchymal compartment of both mild to moderate and severe COPD has been reported (Eurlings et al., 2014); however, the precise contribution of individual collagen subtypes to this change is unclear. The most abundant collagens in airway ECM are fibrillary collagen type I and III and non-fibrillary collagen type IV. In several studies, both increased and reduced collagen I levels in the small airways and parenchyma, both in mild to moderate and in severe COPD have been reported (Kranenburg et al., 2006; Annoni et al., 2012; Eurlings et al., 2014). According to Annoni et al. (2012), the levels of collagen type IV in patients with moderate COPD are comparable to controls. The same was concluded for collagen III, whereas, Harju et al. (2010) report an upregulated expression of the precursor for collagen III in patients with mild to moderate COPD. With the results being controversial, no general conclusion for the expression of collagens in COPD can be drawn. The discrepancy between the studies may be explained by disconcordant behavior of collagen expression in COPD phenotypes, but insufficient data is available to conclude on this aspect. However, given the reduced expression of collagen cross-linking molecules such as decorin and biglycan in severe COPD, the strength of the collagen fibrils may be reduced in COPD (Corsi et al., 2002; Postma and Timens, 2006). Indeed, recent studies using second harmonic generation two-photon microscopy revealed that in COPD the ratio of the number of mature, organized collagen fibrils over the number of immature, disorganized collagen fibrils is lower in COPD than in healthy controls (Tjin et al., 2014).

### Others

For the glycoproteins tenascin and fibronectin increased levels were found in the airways of mild to moderate COPD patients, inversely correlating with lung function of these patients (Liesker et al., 2009; Lofdahl et al., 2011; Annoni et al., 2012). No change in expression was found in the parenchyma (Annoni et al., 2012), although fibronectin gene expression may be reduced in severe COPD (Gosselink et al., 2010). The levels of the polysaccharide hyaluronan in the ECM are upregulated in patients with COPD, and this is considered to be related to local inflammation in the lung airways (Dentener et al., 2005; Eurlings et al., 2014). No major changes in the expression of laminins have been reported (van Straaten et al., 1999; Liesker et al., 2009), although increased expression of laminin β_2_ has been reported in airway smooth muscle of patients with mild to moderate COPD (Kranenburg et al., 2006).

### Summary of ECM changes in COPD

In summary, the extracellular matrix in the airway and parenchymal compartments of patients with COPD is changed in comparison to healthy controls. The most notable and consistently reported changes, relevant to the discussion on airway function, are reductions in the expression or functional organization of elastic fibers and small molecule proteoglycans such as biglycan and decorin in the airways and parenchyma. Some of these changes (notably the changes in elastin) are already visible in mild to moderate COPD and progress toward severe COPD. In addition, fibrosis of the small airways with increased expression of tenascin-C, fibronectin and possibly collagens in patients with mild to moderate COPD can be observed. For severe COPD, these results are more conflicting.

## The time course of biomechanical changes in COPD

In order to properly capture the time course of the biomechanical phenomena occurring in COPD progression, it is necessary to recognize that different time scales are involved in lung mechanics. The biological reaction induced by exposure of ASM cells takes from dozens of seconds to few minutes before an active contraction of the ASM is triggered (Aaron et al., 2012). Then, the elastic response of the extracellular material subjected to the resulting mechanical load occurs instantaneously whereas the viscous response takes an additional dozens of seconds to minutes. Such contractile events occur in addition to the continuous tidal strain imposed by the 12–20 breaths per minute, which is essential for the glassy ASM cells to keep their fluid-like behavior (Krishnan et al., 2008).

Bronchoconstriction induces a temporary stiffening of the smooth muscle and thus the reduction of the tidal stretch of the airway compartment, which temporarily lead (i) the ASM cells to stiffen by reinforcement of their cytoskeleton (An et al., 2006), (ii) the parenchymal ECM to respond (visco)elastically and undergo strain stiffening (Faffe and Zin, 2009) and (iii) the parenchymal cells to temporarily reinforce their actin cytoskeleton for adapting to their new mechanical environment (Trepat et al., 2007). Those phenomena occur within minutes after the contraction. When the ASM relaxes, the cells are properly stretched by the tidal breath again, which helps with recovering their fluid-like behavior (Krishnan et al., 2008). As a consequence, the ECM recovers (visco)elastically its initial mechanical state, also within minutes after dilation of the healthy airway.

In a healthy lung, the mechanical effects on the cells and the ECM are thus expected to be limited in time, so that cells don't require long term mechanical adaptation through ECM remodeling. In contrast, bronchoconstriction is usually more frequent for a longer time in a lung subjected to chronic inflammatory reactions induced by chronic smoke or allergens exposure (Meurs et al., 2008). This increased responsiveness to constricting stimuli is called airway hyper responsiveness (AHR). It is one of the hallmarks of asthma, but is also found in patients with COPD (van den Berge et al., 2012). AHR enhances the frequency and duration of the mechanical loads applied to the cells, which then cope with these changes by remodeling the ECM in their close vicinity (Gosens and Grainge, 2015). In contrast to passive mechanical responses of the lung tissue that are almost immediate (seconds to minutes), active mechanical responses at the tissue scale like remodeling processes are slower (days to weeks) since they involve ECM degradation and formation.

### Chronologic development of COPD

The inflammatory process at the onset of the disease recruits immune cells such as macrophages that secrete various enzymes among which the metallo-elastase (MMP-12) (Lagente et al., 2009). The resulting early degradation of the elastic fibers is potentially the first change of the ECM (Merrilees et al., 2008; Eurlings et al., 2014) and the recoil property of the airway is thus the first mechanical feature to be affected in the course of COPD. Additionally, hyaluronan polysaccharides attract water, which gives the ECM additional resistance to compression (Cavalcante et al., 2005). These changes in the mechanics of airway ECM prevent the full recovery of the original airway caliber when the ASM relaxes after bronchoconstriction (Chung, 2005). Also, elastin fragments resulting from the MMP-12 action further promote the recruitment of macrophages and a first short range vicious circle of ECM remodeling settles in the airway compartment (Lagente et al., 2009). This early sequence of events would already explain the progressive narrowing of the airways and thus the slow degradation of lung function characteristic of the first stage of COPD without obvious emphysema.

These mild airflow limitations being almost imperceptible and therefore usually not treated, both the airway and parenchymal compartments remain mechanically stressed for periods of time sufficient to trigger mechanotransduction pathways in the lung tissue. Indeed, if the smooth muscle has not fully recovered its basal tone, the airway cells are embedded in a new mechanical environment, which they tend to remodel so as to recover a mechanical equilibrium (Humphrey et al., 2014). For example, the long lasting compressive stresses that remain in the airway compartment after a non-fully reversible episode of bronchoconstriction (Brook et al., 2010) would explain an abnormal reinforcement of the ECM by deposition of fibronectin in the small airways in mild to moderate COPD, but also the buckling of the collagen fibers observed by second harmonics generation by Tjin et al. (2014). The increase of tenascin, fibronectin, and possibly collagen expressions in these airways is characteristic of fibrosis, which also contributes to the stiffening of the overall airway wall and thereby further impairs the ability for the embedded ASM cells to relax in the presence of bronchodilators. Therefore, a second vicious circle of ECM remodeling further characterizes the development of the disease in the airway compartment (Suki et al., 2013).

During mild to moderate COPD, mainly the recoil properties of the parenchyma are affected with changes in elastin and hyaluronan expressions (Table 1). Nevertheless, as the airway compartment gets stiffer, an increasing part of the forces generated by the ASM is transmitted to the parenchymal compartment, which progressively gets and remains more and more stretched (Figure 1B). The mechanical properties of the parenchyma are mainly determined by those of the collagenous network subjected to strain stiffening. Even though the results on the evolution of collagen content are somehow conflicting in the literature (Table 1), there is a clear reduction of decorin and biglycan, which insure the formation and cross-linking of collagen fibers. This change in the expression of proteoglycans could be explained by the interstitial cells actively compensating the passive strain stiffening of the collagen fibers by decreasing their crosslinking to soften whole collagenous network. However, these changes not only decrease the rigidity but also the strength of alveolar tissue. When some fibers start to break under a critical loads, part of the stress is released in deformation of the surroundings and part is redistributed to the fibers close by. The amount of interstitial cells in the parenchymal tissue is not sufficient to insure the maintenance of the alveolar ECM that is continuously degraded. Therefore, a third vicious circle of ECM remodeling/degradation characterizes the propagation of the disease in the parenchymal compartment (emphysema) (Suki et al., 2013).

## Implications for future research on COPD

The present review gathers various biomechanical studies performed from the molecular to the organ scale, focusing on the airway and the parenchymal compartments of the lung, and taking into account cellular and extracellular players. Through its integrative point of view on airway mechanics, this article reveals an original perspective on the progression of airway narrowing in COPD. This review indeed suggests that altered ECM composition may affect airway mechanics in COPD, independent of or in addition to parenchymal destruction. Changes in airway wall stiffness, elastic fiber content and composition, and collagen cross-linking molecules such as decorin and biglycan are sufficient to explain changes in airway mechanics in COPD.

The implications of this discussion are significant for studies focusing on airway narrowing and lung repair in COPD. If changes in ECM expression resulting in altered airway wall stiffness and looser parenchymal structure are sufficient to explain at least a part of increased airway narrowing in those mild to moderate COPD patients without obvious emphysema, then it is maybe more prudent to target with drugs the patients in this time specific window during the early progression of COPD. Indeed, damaged tissue is difficult to repair whereas disorganized, yet physically intact tissue may still have a reasonable chance. As a consequence, future studies are clearly needed to estimate the impact of various degrees of ECM damage on airway narrowing. In addition, it would be recommended that investigations focusing on mechanisms of tissue repair incorporate airway mechanics as a read-out to measure the functional impact of the regenerative strategy.

Among the multiple experimental models encountered in the studies cited throughout this review, precision cut lung slices (PCLS) harvested from animals or human donors appear to be well suited to such questions at an intermediate scale (Konigshoff et al., 2011; Sanderson, 2011). Indeed, lung slices can be harvested from healthy or diseased specimen, are viable *in vitro* for several days and provide a natural co-culture, which preserves the native biochemical and mechanical interactions between cells of different types and their ECM. This integrative model of the airway system gives insights in the interplay occurring between the different compartments of the tissue, when contraction and relaxation are chemically induced either at rest (Konigshoff et al., 2011; Ma et al., 2013) or under oscillating stretch to account for the benefits of breathing (Lavoie et al., 2012). Despite the loss of three dimensional structures that may be important for the structural changes occurring during bronchoconstriction, the two dimensional character of the PCLS facilitates the use of many visualization techniques necessary to achieve a reasonable characterization of these changes. Direct comparison between image analysis results and computational models enables the estimation of some properties of the system, not accessible otherwise (Brook et al., 2010). Such computational models supported by experimental evidences are of high interest to simulate the mechanosensing feedback loop between mechanical properties and remodeling in healthy and pathological contexts such as COPD. PCLS constitute therefore a very useful experimental model to explore the consequences of extracellular damage on airway narrowing, but also to test the efficiency of treatments on airway function and understand their action on the airway system as a whole.

## Author contributions

CB, AV and RG each contributed to the first draft of the manuscript and its table and figure. CB, HM and RG revised the draft for important intellectual content. All authors approved the final version of the manuscript.

### Conflict of interest statement

The authors declare that the research was conducted in the absence of any commercial or financial relationships that could be construed as a potential conflict of interest.
